# EGFR inhibition reverses resistance to lenvatinib in hepatocellular carcinoma cells

**DOI:** 10.1038/s41598-022-12076-w

**Published:** 2022-05-14

**Authors:** Xiaoping He, Yohko Hikiba, Yoshimasa Suzuki, Yoshinori Nakamori, Yushi Kanemaru, Makoto Sugimori, Takeshi Sato, Akito Nozaki, Makoto Chuma, Shin Maeda

**Affiliations:** 1 Department of Gastroenterology, Yokohama City University Graduate School of Medicine, 3-9, Fukuura, Kanazawa-ku, Yokohama, 236-0004 Japan; 2 Gastroenterological Center, Yokohama City University Medical Center, Yokohama, Japan

**Keywords:** Cancer, Gastroenterology

## Abstract

Hepatocellular carcinoma (HCC) is a leading cause of cancer-related death worldwide. Lenvatinib is approved as a first-line treatment for unresectable HCC. The therapeutic duration of lenvatinib is limited by resistance, but the underlying mechanism is unclear. To establish lenvatinib-resistant cells, Hep3B cells were initially treated with 3 µM lenvatinib. The concentration was gradually increased by 1 µM or 0.5 µM per week and it reached to 7.5 µM 2 months after the initial exposure to lenvatinib. The biological characteristics of these cells were analyzed by ERK activation in the MAPK signaling pathway and a human phospho‐receptor tyrosine kinase (RTK) antibody array. Factors possibly related to lenvatinib resistance were analyzed using inhibitors, and cell proliferation was analyzed. We established lenvatinib-resistant HCC cells (LR cells) by long-term exposure to lenvatinib. Lenvatinib reduced ERK activation in the parent cells, but not in the LR cells. RTK array analysis showed that the activities of EGFR and insulin-like growth factor 1 receptor (IGF1R)/insulin receptor (INSR) were significantly increased in LR cells, whereas the activities of other RTKs were unchanged. Erlotinib, a widely used EGFR inhibitor, downregulated ERK activation in LR cells. The proliferation of LR cells will also be affected when lenvatinib is combined with erlotinib to treat LR cells. In contrast, inhibition of IGFR/INSR did not affect ERK activation or cell proliferation. Scavenging of reactive oxygen species (ROS) ameliorated the enhanced EGFR activation in LR cells. Lenvatinib resistance was induced by enhanced EGFR activation, possibly via ROS accumulation, in lenvatinib- resistant cells. These findings may enable the development of lenvatinib combination therapies for HCC.

## Introduction

Liver cancer is the second leading cause of cancer death, after lung cancer. It caused approximately 781,000 deaths worldwide in 2018; in that year, hepatocellular carcinoma (HCC) comprised approximately 90% of primary liver cancers^1,2^. Although the incidences of HCC are lower in Europe and the United States than in Asia and Africa, they have gradually increased in recent years^3^. The causes may include non-alcoholic fatty liver disease and non-alcoholic steatohepatitis, as well as hepatitis C^4,5^. Early HCC can be treated by radiofrequency ablation, surgical resection, or liver transplantation, but it has a high recurrence rate and low postoperative survival rate^6–8^. Most patients with liver cancer exhibited advanced disease at the time of diagnosis. Because of their decreased liver function, the above-mentioned treatment options are unsuitable for these patients^9^.

Although the multi-target tyrosine kinase inhibitor, sorafenib, was approved in 2007 as a first-line drug for unresectable HCC and has been used clinically, it has few clinical benefits; moreover, the 5-year relative survival rate of unresectable HCC patients is low after sorafenib treatment^10,11^. As a multi-target tyrosine kinase inhibitor, lenvatinib affects tumor cell proliferation and blood vessel formation by selectively inhibiting receptor tyrosine kinases (RTKs) such as vascular endothelial growth factor receptors 1–3, fibroblast growth factor receptors 1–4, and platelet-derived growth factor receptor α^12^. In the REFLECT trial, the use of lenvatinib as a first-line treatment for advanced HCC demonstrated non-inferiority, compared to sorafenib. This led to the approval of lenvatinib as a front-line treatment for advanced HCC^13^. Because of primary or adaptive drug resistance, the drug resistance of targeted therapy is not negligible, thus hindering the treatment of advanced HCC^14,15^. Accordingly, it is important to explore approaches for overcoming resistance of advanced HCC to lenvatinib by combination therapies or other methods.

Here, we reveal that epidermal growth factor receptor (EGFR) is highly activated in lenvatinib-resistant HCC cells, compared with the parent HCC cells. Treatment of cells with erlotinib, an inhibitor of EGFR, reversed lenvatinib resistance in drug-resistant HCC cells. These data provide evidence for improving or overcoming lenvatinib resistance; they may also provide insights regarding the potential mechanism of lenvatinib resistance.

## Materials and methods

### Materials

HepG2, Hep3B, and Huh-7 HCC cells were acquired from ATCC; JHH-4 and Huh-6 cells were acquired from the JRCB bank. Erlotinib, linsitinib, and lenvatinib were obtained from Selleck. Dulbecco’s Modified Eagle Medium (043-30085) and MEMα (135-15175) were purchased from Wako. Penicillin–streptomycin solution (× 100) (168-23191), 100 mmol/L sodium pyruvate solution (× 100) (190-14881), MEM non-essential amino acids solution (× 100)(139-15651), and 0.25 w/v% trypsin-1 mmol/L EDTA·4Na solution with phenol red (201-16945) were also purchased from Wako.

### Cell culture

HepG2, JHH-4, Huh6, and Huh7 cells were cultured in Dulbecco’s Modified Eagle Medium supplemented with 10% fetal bovine serum, and 1 × penicillin- streptomycin. Hep3B cells were cultured in MEMα supplemented with the above additives, also add 1 × sodium pyruvate, and 1 × MEM non-essential amino acids. The cell lines were maintained in an incubator in a humidified atmosphere containing 5% CO2 at 37 °C.

### Cell proliferation

Cell proliferation was measured by Cell Counting Kit-8 (CCK-8, Dojindo, Japan) assays. In accordance with the standard protocol, 4000 cells were seeded into 96-well palates with four replicates. Next, the cells were treated with lenvatinib and/or linsitinib, or erlotinib alone, for 24–96 h at 37 °C in 5% CO2. Next, 10 µL of CCK-8 solution were added to each well and incubated for 30 min. OD at 450 nm values were determined using a microplate reader. IC50 values were defined by non-linear regression (curve fit) analysis using GraphPad Prism 9.3.1 (350) Macintosh Version by Software MacKiev © 1994–2021 GraphPad Software, LLC for macOS.

### Development of lenvatinib-resistant cell lines

To establish lenvatinib-resistant cells, parental cells were treated with 3 µM lenvatinib. The lenvatinib concentration was increased by 1 or 0.5 µM per week, reaching 7.5 µM after 2 months of initial exposure. Finally, a lenvatinib-resistant (LR) cell line was established. LR cells were continuously maintained in the presence of 7.5 µM lenvatinib.

### Immunoblotting

Cells were washed and lysed with 2 × sodium dodecyl sulfate sample buffer. Next, proteins were separated by 5–20% sodium dodecyl sulfate–polyacrylamide gel electrophoresis, then transferred to a polyvinylidene difluoride membrane. The membrane was incubated with the indicated primary antibodies at 4 °C overnight. After incubation with a horseradish peroxidase-linked secondary antibody, immunoreactive bands were detected using an imaging system (ChemiDoc Touch). The primary antibodies were an anti-EGFR antibody (A11351; ABclonal), anti-β-actin (ACTB) antibody (9126; Cell Signaling Technology), anti-β-tubulin (TUBB) antibody (014-25041; Wako), anti-p44/42 MAPK (Erk1/2) antibody (#4695; Cell Signaling Technology), anti-phospho- p44/42 MAPK (Erk1/2) antibody (#4370; Cell Signaling Technology), anti-phospho-EGFR antibody (Y1068) (AP0301; ABclonal), and anti-phospho-insulin receptor antibody (AP0046; ABclonal). And the protein bands were semi-quantification using Image J software with p-protein/total protein or p-protein/internal control ratios (Rasband, W.S., ImageJ, U. S. National Institutes of Health, Bethesda, Maryland, USA, https://imagej.nih.gov/ij/, 1997–2018).

### Human phospho‐RTK antibody array

Hep3B cells and lenvatinib-resistant cells (LR7.5-3B) cells were treated with vehicle or lenvatinib. The cells were harvested and lysed with lysis buffer containing a protease and phosphatase inhibitor cocktail. Protein quantification was performed using a Standard BCA Protein Assay Kit (Thermo Scientific, Fremont, CA, USA), followed by analysis using a Human Phospho-RTK Antibody Array Kit (ARY001B; R&D Systems), in accordance with the manufacturer’s instructions. Array images were visualized by a chemiluminescence detection kit (Millipore, Billerica, MA, USA) and analyzed by Image Lab Software (Bio‐Rad).

### RT-qPCR

Hep3B and LR7.5-3B cells were treated with vehicle or 3 μM lenvatinib for 4 h. Isogen with a Spin Column (314-07513) from Nippon Gene was used to extract total RNA. cDNA synthesis was performed using ReverTra Ace qPCR RT Master Mix (FSQ-201) from Toyobo. The qPCR reaction was prepared using KOD SYBR qPCR Mix (QKD-201T) also from Toyobo. Experiments were performed in accordance with the manufacturer’s instructions.

### Detection of ROS

To quantitatively assess reactive oxygen species (ROS), we used the 2′,7′-dichlorofluorescin diacetate (DCFH-DA) assay, which is based on the diffusion of DCFH-DA into cells. The diffused DCFH-DA is deacetylated by cellular esterases to produce a non-fluorescent compound, which is oxidized by ROS into 2′,7′-dichlorofluorescein (DCF). DCF is highly fluorescent and can be detected by fluorescence spectroscopy with excitation/emission at 485/535 nm.

### Statistical analysis

Data are expressed as the mean ± standard deviation (SD). Significant differences were determined using Student's t-test. *p* values ≤ 0.05 were considered significant.

### Consent to publish

All authors contributed to the interpretation of the data and reviewed and approved the manuscript.

## Results

### Effect of lenvatinib on the MAPK signaling pathway

**Figure 1:**
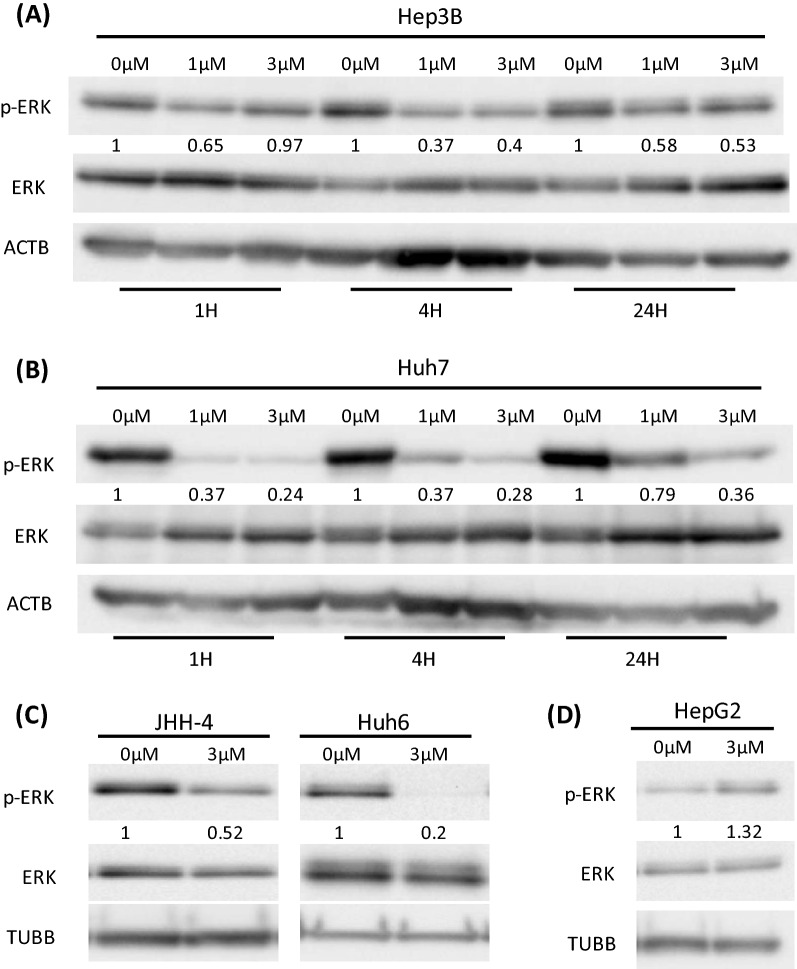
Effects of lenvatinib on the MAPK signaling pathway in HCC cells. (**A**,**B**) Lenvatinib was added at concentrations of 0, 1, and 3 μM in medium containing 10% fetal bovine serum for 1, 4, and 24 h, respectively. Lenvatinib downregulated ERK phosphorylation in Hep3B cells (**A**) and Huh7 cells (**B**). (**C**) Lenvatinib at 3 µM for 4 h downregulated ERK phosphorylation in JHH-4 and Huh6 cells. (**D**) Lenvatinib at 3 µM for 4 h did not downregulate ERK phosphorylation in HepG2 cells. In all experiments, immunoblotting of phosphorylated ERK (p-ERK), ERK, and internal controls (ACTB or TUBB) levels was performed. The intensity with the internal control were indicated. At least two times of the independent experiments of (**A**-**D**) were performed and representative results were shown.

Most RTK-mediated signaling activates the mitogen-activated protein kinase (MAPK) cascade, comprising the Raf, MEK, and extracellular signal-regulated kinase (ERK). Therefore, we examined the effects of lenvatinib on ERK activation in Hep3B, Huh7, JHH-4, Huh6, and HepG2 HCC cells by immunoblotting analysis. Treatment with lenvatinib at 0,1, and 3 μM for 1,4, and 24 h significantly downregulated ERK phosphorylation in Hep3B and Huh7 cells (Fig. 1A,B). We also observed inhibition in JHH-4 and Huh6 cells that had been treated with 3 µM lenvatinib for 4 h (Fig. 1C). When HepG2 cells were treated with 3 µM lenvatinib for 4 h, ERK phosphorylation was not inhibited (Fig. 1D). Therefore, lenvatinib significantly downregulated the phosphorylation of ERK in the MAPK signaling pathway in most HCC cell lines (Fig. 1A–D).

### Establishment of a lenvatinib-resistant HCC cell line

**Figure 2:**
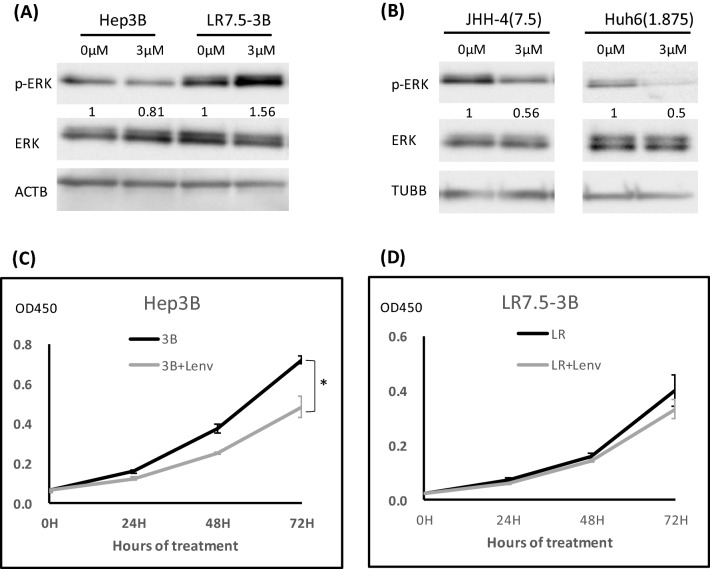
Establishment of lenvatinib-resistant cells. (**A**) Lenvatinib-resistant Hep3B cells were established by treatment with lenvatinib for > 2 months (LR7.5-3B). Immunoblotting analysis of phosphorylated ERK (p-ERK), ERK, and ACTB levels in Hep3B and LR7.5-3B cells with or without 3 μM lenvatinib for 4 h. (**B**) Huh6 and JHH-4 cells were treated with 1.875 and 7.5 μM lenvatinib, respectively, for > 1 month. After treatment of the above-mentioned Huh6 and JHH-4 cells with 3 µM lenvatinib for 4 h, phosphorylation levels of ERK (p-ERK), ERK and TUBB were analyzed by immunoblotting. The intensity with the internal control were indicated. (**C**,**D**) Cell proliferation was analyzed by Cell Counting Kit 8 (CCK-8) assays in normal (Hep3B) and lenvatinib-resistant (LR7.5-3B) Hep3B cells that had been treated with 3 μM lenvatinib at the indicated times (n = 4 per treatment). Three independent experiments of (**A**–**D**) were performed, and representative results are shown. *p < 0.05.

To explore the mechanism of HCC resistance to lenvatinib, parental cells were initially treated with 3 µM of lenvatinib. The concentration was gradually increased by 1 µM or 0.5 µM per week and it reached to 7.5 µM. When the HCC cell line was able to tolerate a higher dose of lenvatinib (cell proliferation was not inhibited by lenvatinib for 72 h), compared with the parent cells, we regarded this as successful generation of a resistant Hep3B cell line (LR7.5-3B); this process required 2 months of culture. In LR7.5-3B cells, lenvatinib enhanced ERK phosphorylation, rather than the initially observed inhibition (Fig. 2A). The IC50 of Hep3B and LR7.5-3B were 2.8 µM and > 30 µM, respectively (Fig. S1). We were unable to establish lenvatinib-resistant, JHH-4 and Huh6 cells (Fig. 2B). Lenvatinib did not inhibit the proliferation of LR7.5-3B cells, whereas it significantly inhibited the proliferation of Hep3B cells (Fig. 2C,D). Therefore, Hep3B and LR7.5-3B cells were used in subsequent analyses.

### Enhanced activation of EGFR and IGF1R/INSR in lenvatinib-resistant cells

**Figure 3:**
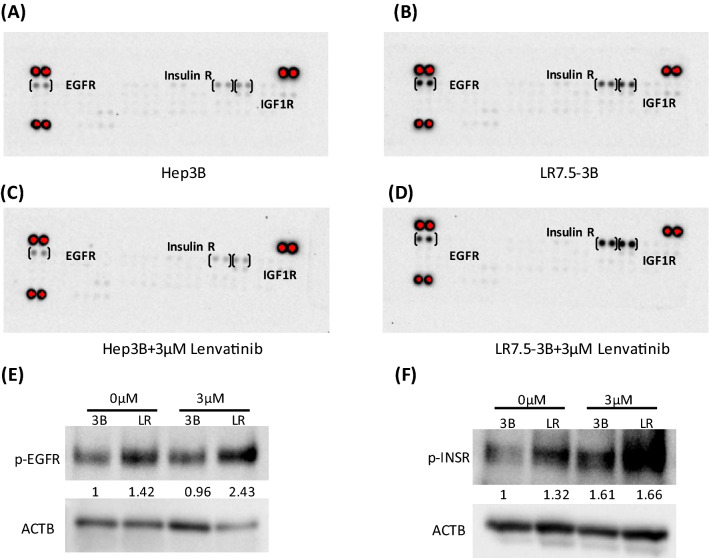
Phosphorylation levels of EGFR and IGF1R/INSR were higher in lenvatinib-resistant cells. (**A**–**D**) The Proteome Profiler Human Phospho-RTK Array Kit was used to determine the relative levels of tyrosine phosphorylation of human receptor tyrosine kinases (RTKs) in Hep3B cells (**A**), LR7.5-3B cells (**B**), Hep3B cells treated with 3 µM lenvatinib for 4 h (**C**), and LR7.5-3B cells treated with 3 µM lenvatinib for 4 h (**D**). (**E**) Immunoblotting analysis of phosphorylated EGFR (p-EGFR) and ACTB levels in Hep3B (3B) and LR7.5-3B (LR) cells that had been treated with or without 3 μM lenvatinib for 4 h. (**F**) Immunoblotting analysis of phosphorylated INSR (p-INSR) and ACTB levels in Hep3B (3B) and LR7.5-3B (LR) cells that had been treated with or without 3 μM lenvatinib for 4 h. The intensity with the internal control were indicated. Two independent experiments of (**E**,**F**) were performed, and representative results are shown.

Using the Proteome Profiler Human Phospho-RTK Array Kit, we found that the phosphorylation levels of EGFR and insulin-like growth factor 1 receptor (IGF1R)/insulin receptor (INSR) were higher in LR7.5-3B cells than in Hep3B cells (Fig. 3A,B). These results were confirmed by immunoblotting analysis (Fig. 3E,F). Lenvatinib slightly reduced the activation of EGFR and IGF1R/INSR in Hep3B cells (Fig. 3A,C), but it did not affect or slightly increased the activation levels of these receptors in LR7.5-3B cells (Fig. 3B,D).

### Erlotinib inhibits the phosphorylation of ERK and reverses resistance to lenvatinib

**Figure 4:**
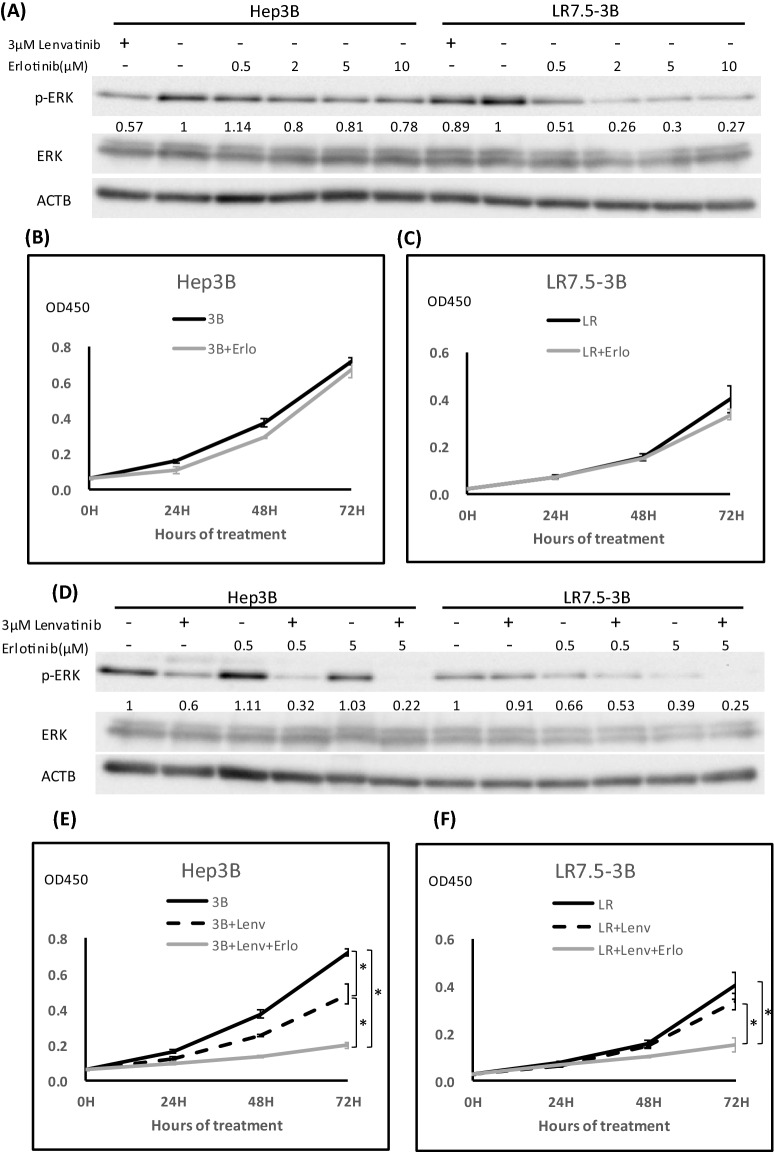
Effects of the EGFR inhibitor erlotinib on ERK phosphorylation and cell proliferation. (**A**) Immunoblotting analysis of phosphorylated ERK (p-ERK), ERK , and ACTB levels in Hep3B and LR7.5-3B cells that had been treated with 3 μM lenvatinib or the indicated concentrations of erlotinib for 4 h. (**B**, **C**) Cell proliferation was analyzed by Cell Counting Kit 8 (CCK-8) assays in Hep3B (3B) (**B**) and LR7.5-3B (LR) (**C**) cells that had been treated with or without 0.5 μM erlotinib at the indicated times (n = 4 per treatment). Representative results are shown. (**D**) Immunoblotting analysis of phosphorylated ERK (p-ERK), ERK and ACTB levels in Hep3B and LR7.5- 3B cells that had been treated with or without 3 μM lenvatinib, alone or combined with 0.5 or 5 μM erlotinib for 4 h. (**E**,**F**) Cell proliferation was analyzed by Cell Counting Kit 8 (CCK-8) assays in Hep3B (3B) (**E**) and LR7.5-3B (LR) (**F**) cells that had been treated with or without 3 μM lenvatinib, alone or combined with 0.5 μM erlotinib, at the indicated times (n = 4 per treatment). The intensity with the internal control were indicated. Two independent experiments of were performed, and representative results are shown. *p < 0.05.

EGFR activation was increased in LR7.5-3B cells, compared with their parent cells; therefore, we tested the effects of EGFR inhibition. Erlotinib, as an inhibitor of the tyrosine kinase receptor EGFR (Fig. S6), is used to treat non-small cell lung cancer and pancreatic cancer^16,17^. ERK activation was slightly attenuated in Hep3B cells upon treatment with erlotinib alone. In contrast, increased ERK activation in LR7.5-3B cells was inhibited by erlotinib in a dose-dependent manner (Fig. 4A). To measure the proliferation of the cells, we used 0.5 µM erlotinib, because this concentration is started to inhibit ERK activity in LR7.5-3B. Erlotinib (0.5 µM) alone did not inhibit the proliferation of Hep3B or LR7.5-3B cells (Fig. 4B,C). Erlotinib and lenvatinib synergistically reduced ERK phosphorylation in Hep3B and LR7.5-3B cells (Fig. 4D). Furthermore, the proliferation of LR7.5-3B cells was inhibited by combination treatment (Fig. 4E,F), but it was not inhibited by lenvatinib alone (Fig. 2D). Therefore, enhanced EGFR activation is involved in lenvatinib resistance in LR7.5- 3B cells, and it can be reversed by the addition of erlotinib.

### The IGF1R/INSR inhibitor linsitinib did not affect ERK phosphorylation or cell proliferation

**Figure 5:**
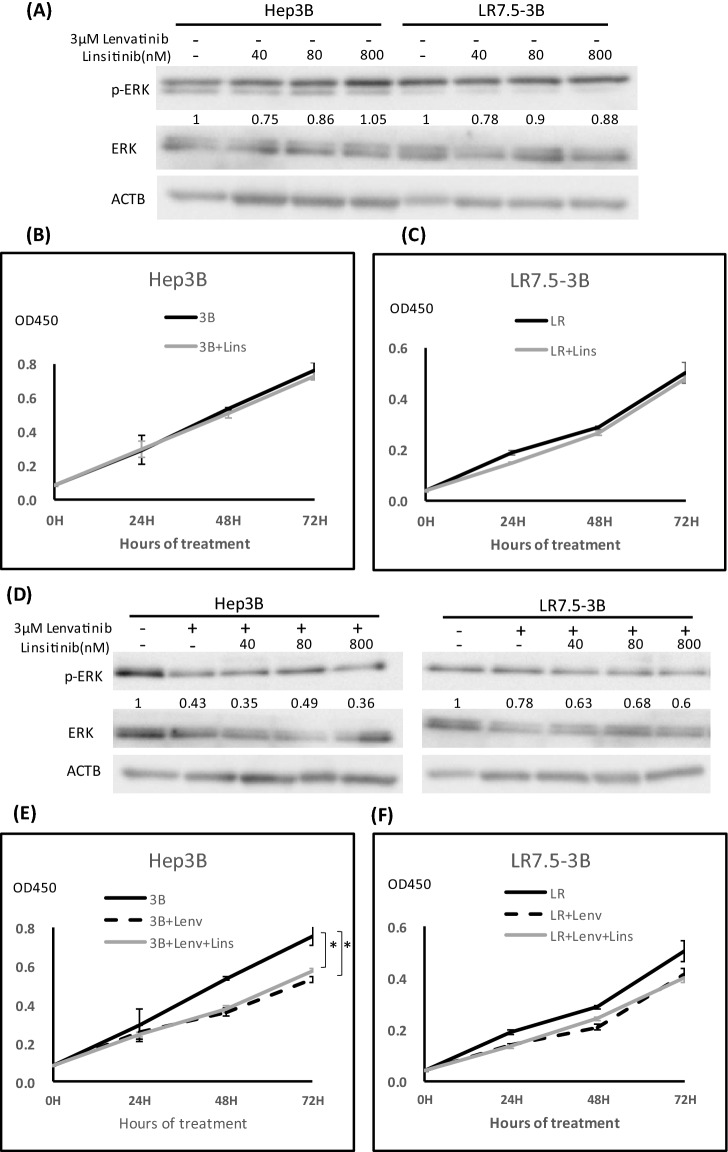
Effect of the IGF1R inhibitor linsitinib on ERK phosphorylation and cell proliferation. (**A**) Immunoblotting analysis of phosphorylated ERK (p-ERK), ERK and ACTB levels in Hep3B and LR7.5- 3B cells that had been treated with linsitinib for 4 h. (**B**,**C**) Cell proliferation was analyzed by Cell Counting Kit 8 (CCK-8) assays in Hep3B (3B) (**B**) and LR7.5-3B (LR) (**C**) cells that had been treated with or without 0.1 μM linsitinib at the indicated times (n = 4 per treatment). Representative results are shown. (**D**) Immunoblotting analysis of phosphorylated ERK (p-ERK), ERK and ACTB levels in Hep3B and LR7.5-3B cells that had been treated with or without 3 μM lenvatinib, alone or combined with the indicated concentration of linsitinib for 4 h. (**E**,**F**) Cell proliferation was analyzed by Cell Counting Kit 8 (CCK-8) assay in Hep3B (3B) (**E**) and LR7.5-3B (LR) (**F**) cells treated with or without 3 μM lenvatinib, alone or combined with 0.1 µM linsitinib, at the indicated times (n = 4 per treatment). The intensity with the internal control were indicated. Two independent experiments were performed, and representative results are shown. *p < 0.05.

Linsitinib, as an inhibitor of INSR and IGF1R, is often used as an experimental drug candidate for the treatment of various types of cancer^18^ (Fig. S6). Linsitinib alone did not inhibit ERK phosphorylation or proliferation in Hep3B and LR7.5-3B cells (Fig. 5A–C). Linsitinib and lenvatinib in combination did not affect ERK phosphorylation in LR7.5-3B cells (Fig. 5D). Additionally, cell proliferation was not inhibited by the addition of linsitinib (Fig. 5E,F). Therefore, enhanced INSR and IGF1R activation was not involved in lenvatinib resistance in LR7.5-3B cells.

### Increased ROS production has a critical role in EGFR/ERK activation in lenvatinib-resistant cells

**Figure 6:**
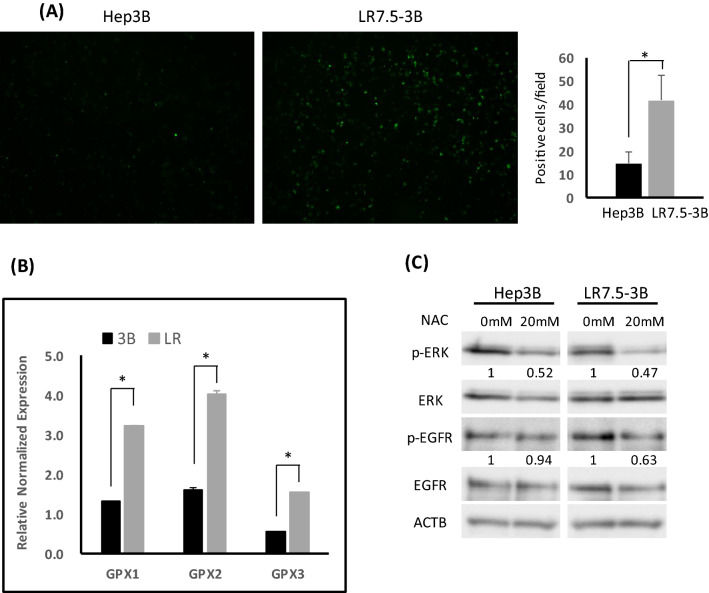
Involvement of ROS in EGFR and ERK activation in lenvatinib-resistant cells. (**A**) Hep3B and LR7.5-3B cells were treated with DCFH-DA for 30 min on the day of use, then washed twice with the same amount of Hank’s Balanced Saline Solution ( +); ROS content was analyzed using the all-in-one fluorescence microscope BZ-X800. Graphs were average of positive cells in the 12 field. (**B**) Real-time PCR analysis of the glutathione peroxidase (GPX1-3) gene in Hep3B (3B) and LR7.5-3B (LR) cells. Two independent experiments of were performed, and representative results are shown. (**C**) Immunoblotting analysis of phosphorylated ERK (p-ERK), ERK, phosphorylated EGFR (p-EGFR), EGFR and ACTB levels in Hep3B and LR7.5-3B cells that had been treated with or without 20 mM NAC for 1 h. The intensity with the internal control were indicated. Two independent experiments were performed, and representative results are shown. *p < 0.05.

Reactive oxygen species (ROS) are byproducts of aerobic metabolism and are related to oxidative stress^19^. Endogenous ROS elevation can increase EGFR autophosphorylation^20,21^. Therefore, to analyze the involvement of ROS in the increased EGFR activation in resistant cells, we assayed the ROS content in Hep3B and LR7.5-3B cells with DCFH-DA assays. The ROS content was higher in LR7.5-3B cells than in Hep3B cells (Fig. 6A). Furthermore, Gpx1, Gpx2, and Gpx3 mRNA expression levels were increased in LR7.5-3B cells, compared with Hep3B cells, suggesting that the increased ROS production induced antioxidant enzyme expression to scavenge ROS (Fig. 6B). The antioxidant N-acetylcysteine (NAC) is used for investigating ROS in biological processes^22^. NAC inhibited ERK and EGFR phosphorylation in LR7.5-3B cells; in Hep3B cells, NAC slightly inhibited ERK phosphorylation and did not inhibit EGFR phosphorylation (Fig. 6C). Therefore, increased ROS production may have a key role in the activation of EGFR/ERK in lenvatinib-resistant cells.

## Discussion

Although sorafenib has been approved as a first-line treatment for HCC, its clinical benefit is not significant^10,11^. Compared with sorafenib, lenvatinib has demonstrated non-inferiority and can improve clinical efficacy, although its median overall survival is limited to approximately 1 year^13^. Patients with advanced HCC may develop resistance to lenvatinib^14,15^. To explore the mechanisms underlying acquired lenvatinib resistance and the recovery of lenvatinib sensitivity, we developed a human HCC cell line with lenvatinib resistance via long-term exposure to lenvatinib.

Enhanced EGFR activation is a mechanism of lenvatinib resistance. In this study, we found that only Hep3B cells were amenable to the creation of resistant lines. This is presumably because many cells are resistant to lenvatinib before treatment, hampering the creation of a resistant strain. Indeed, the activation level of EGFR is reportedly related to susceptibility to lenvatinib^23^.

The activation of other RTKs comprises a mechanism of tyrosine kinase inhibitor resistance. This feedback system provides common signals (e.g., MAPK, downstream of RTK) that facilitate the survival of cancer cells. Various factors may be involved, such as gene mutations and epigenetic alterations^24^. In this study, an RTK array was used to screen for other RTK activations in resistant cells. The activated states of EGFR and IGF1R were consistently enhanced. Notably, EGFR inhibition reversed lenvatinib resistance and reduced ERK activation in resistant cells. Therefore, lenvatinib-resistant cells may have increased dependence on EGFR, compared with their parent cells.

The EGFR system is a “signal hub” where extracellular growth and survival signals converge^25^. EGFR is important in cell proliferation, survival, and migration^25^. EGFR positivity is observed in 68% of HCC patients, and its overexpression is related to aggressive tumors, metastasis, and poor survival^26,27^. In HCC, activation of the downstream MAPK/ERK signaling pathway promotes cell proliferation, movement, and survival; it also supports tumor progression by promoting tumor growth, invasiveness, and angiogenesis^28,29^.

Erlotinib alone decreased ERK activation in LR7.5-3B, but the cell proliferation was not inhibited by erlotinib alone. It was very interesting. We suppose that ERK activation is an important marker for the proliferation. However, in addition to ERK activation, other than ERK activation is necessary for the cell proliferation. So far, we could not identify the putative factors. In lenvatinib-sensitive cells, lenvatinib may inhibit ERK/MAPK as well as other putative factors, whereas in the resistant cells, both erlotinib and lenvatinib inhibited ERK/MAPK, but may be still need to lenvatinib for inhibition the putative factors (Fig. S7).

One of the putative mechanisms for increased EGFR activation in lenvatinib-resistant cells is that autocrine production of EGFR ligand is increased. Thus, we measured the mRNA levels of EGFR ligand, such as EGF, TGFα, HB-EGF, EREG and EPGN, in Hep3B and LR7.5-3B cells. but the mRNA expression levels of EGF, TGFα, and HB-EGF were very small, and no difference was found between lenvatinib sensitive (Hep3B) and resistant cells (LR7.5-3B). Although mRNA expression levels of EREG and EPGN were detectable with higher amount, there was no difference between Hep3B and LR7.5-3B. According to those results, we concluded the autocrine production was not clear in the resistant cells. Our findings suggest that ROS cause enhanced EGFR activation in lenvatinib-resistant cells. Additional experiments (data not shown) involving RNA-seq analysis indicated changes in the expression levels of glutathione peroxidases, which remove ROS; these changes were verified by real-time PCR. The ROS levels were increased in resistant cells; removal of ROS by NAC led to reduced EGFR activation. EGFR is activated by ROS via oxidation^20^. The mechanism by which lenvatinib increases intracellular ROS is unclear.

Increased IGF1R activation by resistant cells was also observed in this study. Linsitinib, an inhibitor of INSR and IGF1R, is currently used as an experimental drug candidate for the treatment of various types of cancer^18^. The direct involvement of IGF1R in linsitinib resistance is unclear because this resistance could not be reversed. IGF1R is activated when tyrosine kinase inhibitors are used in lung cancer^30^, which may lead to resistance. When EGFR inhibition occurs in patients with lenvatinib-resistant HCC, IGF1R may be involved in the acquisition of further resistance.

In conclusion, the expression levels of RTKs—EGFR, IGF1R, and InsulinR—were increased in lenvatinib- resistant Hep3B cells. In addition, erlotinib downregulated abnormally activated ERK in lenvatinib-resistant Hep3B cells. When used in combination with lenvatinib, erlotinib restored lenvatinib sensitivity to drug-resistant HCC cell lines. These findings suggest a potential combination therapy approach for HCC.

## Supplementary Information

Supplementary Figures.

## Data Availability

Authors can confirm that all relevant data are included in the article. We agree with the policy in the journal.

## Abbreviations

ACTB: β-Actin
EGFR: Epidermal growth factor receptor
IGF1R: Insulin-like growth factor 1 receptor
INSR: Insulin receptor
NAC: N-acetylcysteine
ROS: Reactive oxygen species
RTK: Receptor tyrosine kinase
TUBB: β-Tubulin

## References

[1] Bray F, Ferlay J, Soerjomataram I, Siegel RL, Torre LA, Jemal A (2018). Global cancer statistics 2018: GLOBOCAN estimates of incidence and mortality worldwide for 36 cancers in 185 countries. CA Cancer J. Clin..

[2] Zhou M, Wang H, Zeng X, Yin P, Zhu J (2019). Mortality, morbidity, and risk factors in China and its provinces, 1990–2017: A systematic analysis for the Global Burden of Disease Study 2017. Lancet.

[3] Baecker A, Liu X, La Vecchia C, Zhang ZF (2018). Worldwide incidence of hepatocellular carcinoma cases attributable to major risk factors. Eur. J. Cancer Prev..

[4] Petrick JL, Kelly SP, Altekruse SF, McGlynn KA, Rosenberg PS (2016). Future of hepatocellular carcinoma incidence in the United States forecast through 2030. J. Clin. Oncol..

[5] Klevens RM, Liu S, Roberts H, Jiles RB, Holmberg SD (2014). Estimating acute viral hepatitis infections from nationally reported cases. Am. J. Public Health.

[6] Llovet JM, Lencioni R, Di Bisceglie AM, Gaile PR, Dufour JF, Greten TF (2012). EASL-EORTC clinical practice guidelines: Management of hepatocellular carcinoma. J. Hepatol..

[7] Zheng J, Chou JF, Gonen M, Vachharajani N, Chapman WC, Doyle MBM (2017). Prediction of hepatocellular carcinoma recurrence beyond Milan Criteria after resection: Validation of a clinical risk score in an international cohort. Ann. Surg..

[8] Famularo S, Di Sandro S, Giani A, Sandini M, De Carlis R, Buscemi V (2018). Recurrence patterns after anatomic or parenchyma-sparing liver resection for hepatocarcinoma in a western population of cirrhotic patients. Ann. Surg. Oncol..

[9] Lin SB, Hoffmann K, Schemmer P (2012). Treatment of hepatocellular carcinoma: A systematic review. Liver Cancer.

[10] Llovet JM, Ricci S, Mazzaferro V, Hilgard P, Gane E, Blanc JF (2008). Sorafenib in advanced hepatocellular carcinoma. N. Engl. J. Med..

[11] Llovet JM, Zucman-Rossi J, Pikarsky E, Sangro B, Schwartz M, Sherman M (2016). Hepatocellular carcinoma. Nat. Rev. Dis. Primers.

[12] Yamamoto Y, Matsui J, Matsushima T, Miyazaki K, Nakamura K, Tohyama O (2014). Lenvatinib, an angiogenesis inhibitor targeting VEGFR/FGFR, shows broad antitumor activity in human tumor xenograft models associated with microvessel density and pericyte coverage. Vasc Cell.

[13] Kudo M, Finn RS, Qin S, Han KH, Ikeda K, Piscaglia F (2018). Lenvatinib versus sorafenib in first-line treatment of patients with unresectable hepatocellular carcinoma: A randomized phase 3 non-inferiority trial. Lancet.

[14] Zhu YJ, Zheng B, Wang HY, Chen L (2017). New knowledge of the mechanisms of sorafenib resistance in liver cancer. Acta Pharmacol. Sin..

[15] Fu R, Jiang S, Li J, Chen H (2020). Activation of the HGF/c-MET axis promotes lenvatinib resistance in hepatocellular carcinoma cells with high c-MET expression. Med. Oncol..

[16] Shepherd FA, Pereira JR, Ciuleanu T, Tan EH, Hirsh V, Thongprasert S (2005). Erlotinib in previously treated non-small-cell lung cancer. N. Engl. J. Med..

[17] Moore MJ, Goldstein D, Hamm J, Figer A, Hecht JR, Gallinger S (2007). Erlotinib plus gemcitabine compared with gemcitabine alone in patients with advanced pancreatic cancer: A phase III trial of the National Cancer Institute of Canada clinical trials group. J. Clin. Oncol..

[18] Mulvihill MJ, Cooke A, Rosenfeld-Franklin M, Buck E, Foreman K, Landfair D (2009). Discovery of OSI- 906: A selective and orally efficacious dual inhibitor of the IGF-I receptor and insulin receptor. Future Med. Chem..

[19] Cross CE, Halliwell B, Borish ET, Pryor WA, Ames BN, Saul RL (1987). Oxygen radicals and human disease. Ann. Intern. Med..

[20] Corcoran A, Cotter TG (2013). Redox regulation of protein kinases. FEBS J..

[21] Heppner DE, van der Vliet A (2016). Redox-dependent regulation of epidermal growth factor receptor signaling. Redox Biol..

[22] Zafarullah M, Li WQ, Sylvester J, Ahmad M (2003). Molecular mechanisms of N-acetylcysteine actions. Cell. Mol. Life Sci..

[23] Jin HJ, Shi YP, Lv YY, Yuan SX, Ramirez CFA, Lieftink C (2021). EGFR activation limits the response of liver cancer to lenvatinib. Nature.

[24] Saraon P, Pathmanathan S, Snider J, Lyakisheva A, Wong V, Stagljar I (2021). Receptor tyrosine kinases and cancer: Oncogenic mechanisms and therapeutic approaches. Oncogene.

[25] Arteaga CL, Engelman JA (2014). ERBB receptors: From oncogene discovery to basic science to mechanism-based cancer therapeutics. Cancer Cell.

[26] Kira S, Nakanishi T, Suemori S, Kitamoto M, Watanabe Y, Kajiyama G (1997). Expression of transforming growth factor alpha and epidermal growth factor receptor in human hepatocellular carcinoma. Liver.

[27] Ito Y, Takeda T, Sakon M, Tsujimoto M, Higashiyama S, Noda K (2001). Expression and clinical significance of erb-B receptor family in hepatocellular carcinoma. Br. J. Cancer.

[28] Jean PG, Christophe F, Georges B (2012). MAPK MEK1/2-ERK1/2 pathway and its implication in hepatocyte cell cycle control. Int. J. Hepatol..

[29] Delire B, Stärkel P (2015). The Ras/MAPK pathway and hepatocarcinoma: Pathogenesis and therapeutic implications. Eur. J. Clin. Investig..

[30] Hayakawa D, Takahashi F, Mitsuishi Y, Tajima K, Hidayat M, Winardi W (2020). Activation of insulin-like growth factor-1 receptor confers acquired resistance to osimertinib in non-small cell lung cancer with EGFR T790M mutation. Thorac. Cancer.

