# Supplementary material for: EGFR inhibition reverses resistance to lenvatinib in hepatocellular carcinoma cells
Source: Sci Rep. 2022 May 14;12:8007. doi: 10.1038/s41598-022-12076-w (PMC9107466; doi:10.1038/s41598-022-12076-w)
Supplement: Supplementary file 1 — Supplementary Figures. [file 41598_2022_12076_MOESM1_ESM.pdf]

EGFR inhibition reverses resistance to lenvatinib in hepatocellular carcinoma cells

Supporting information

Figure S1. IC50 of Hep3B, LR7.5-3B and HepG2 by lenvatinib treatment.

In vitro 24 h anti-proliferative activity of lenvatinib in three human HCC cell lines, Hep3B, HepG2 and lenvatinib-resistant (LR)cells. The IC50 of Hep3B and LR cells were 2.8μM and>30μM,respectively. We also analyzed HepG2 cells, but IC50 was>39μM.

Figure S2-5.

Western blot analysis of the complete blot image, together with the recording of the calculation of the p-protein/total protein ratio in a semi-quantitative manner.

Figure S6.

The inhibition of EGFR or IGF1R by erlotinib or linstinib treatment.

Figure S7.

Putative mechanism for lenvatinib resistance in Hep3B cells.

Figure S1. Determination of inhibitory concentrations at 50% (IC50) values for Lenvatinib in HCC cell lines incubated for 24 hours.

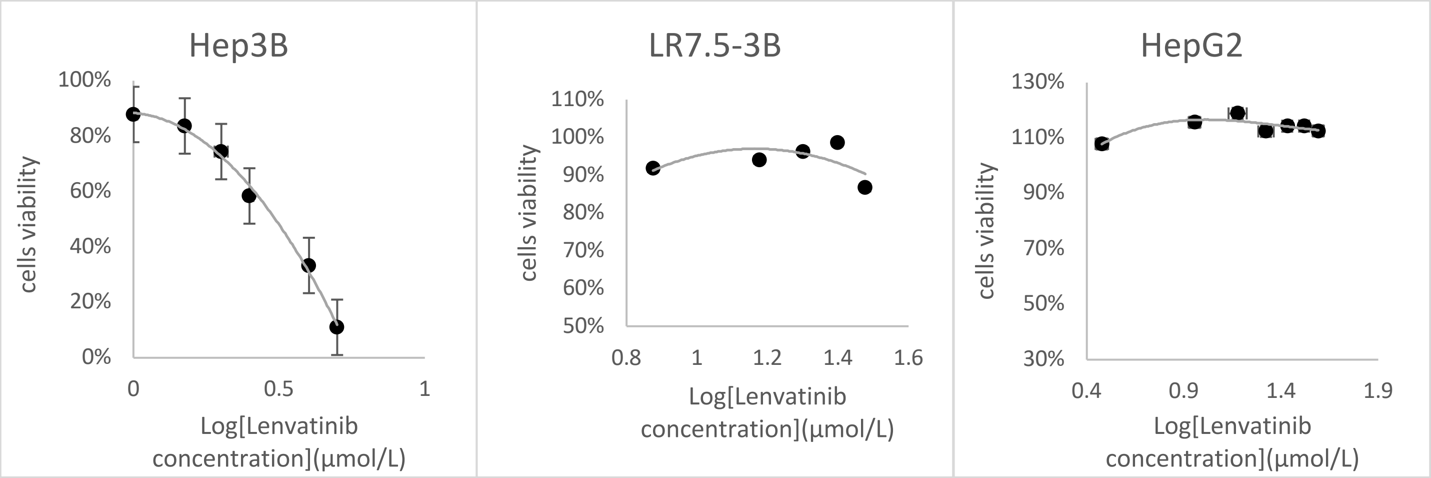

In vitro 24 h anti-proliferative activity of lenvatinib in three human HCC cell lines, Hep3B, HepG2 and lenvatinib-resistant (LR)cells. The IC50 of Hep3B and LR cells were 2.8μM and>30μM,respectively. We also analyzed HepG2 cells, but IC50 was>39μM.

<u>Figure S2-5. Full blot images of Western blotting assay</u>

Figure1(A) Hep3B Semi-quantification of p-ERK/ERK was performed using Image J

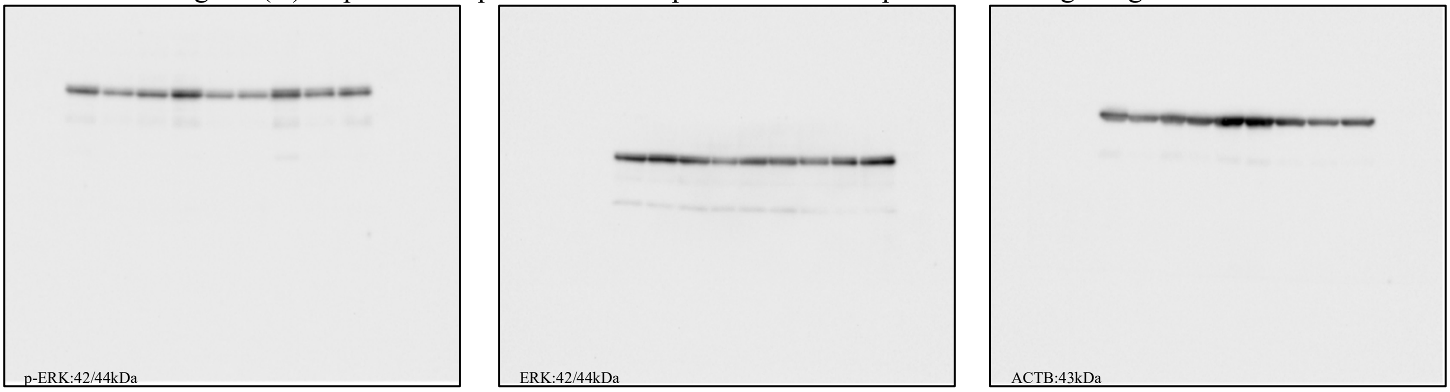

Figure1(B) Huh7 Semi-quantification of p-ERK/ACTB was performed using Image J

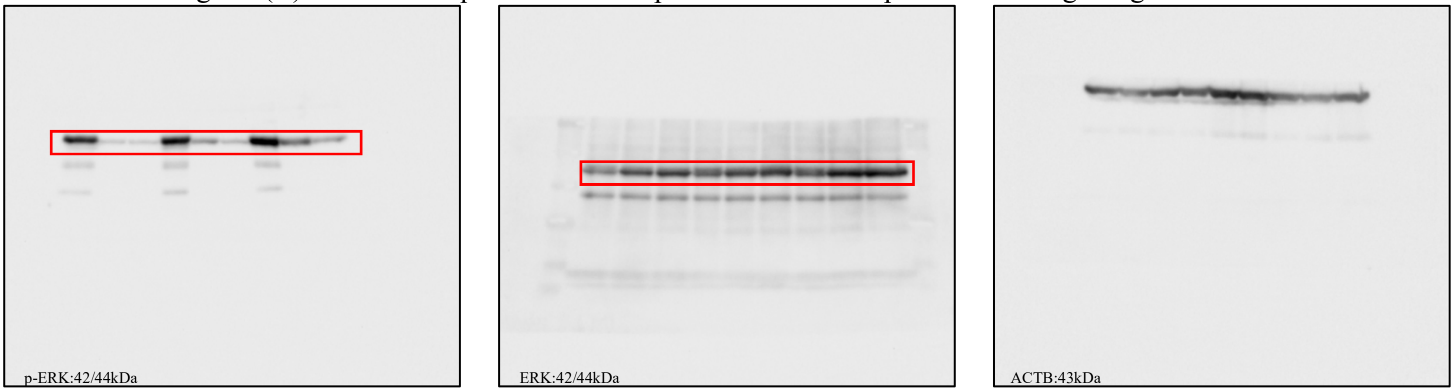

Figure1(C) JHH-4 Semi-quantification of p-ERK/TUBB was performed using Image J

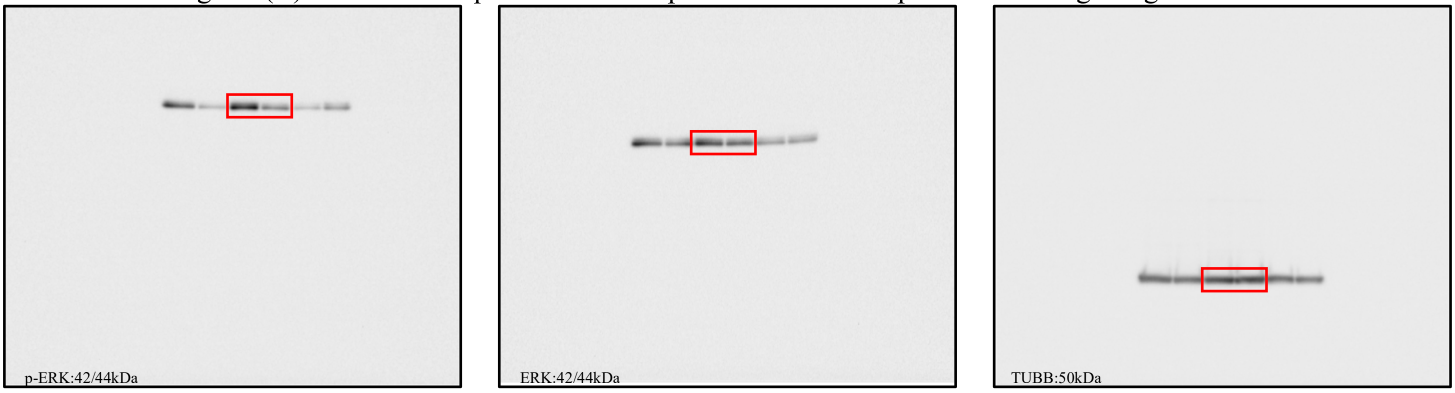

Figure1(C) Huh6 Semi-quantification of p-ERK/TUBB was performed using Image J

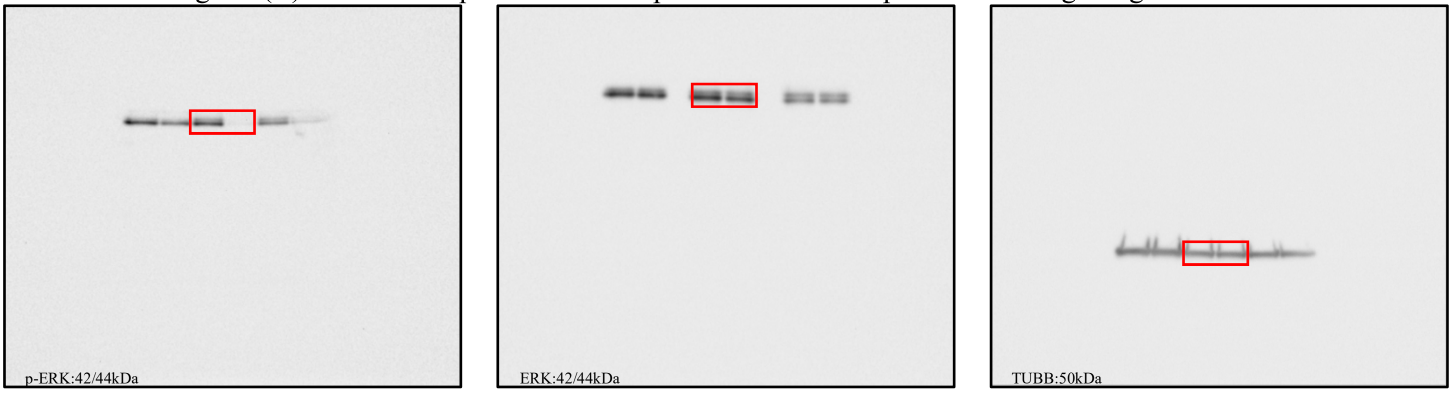

Figure1(D) HepG2 Semi-quantification of p-ERK/TUBB was performed using Image J

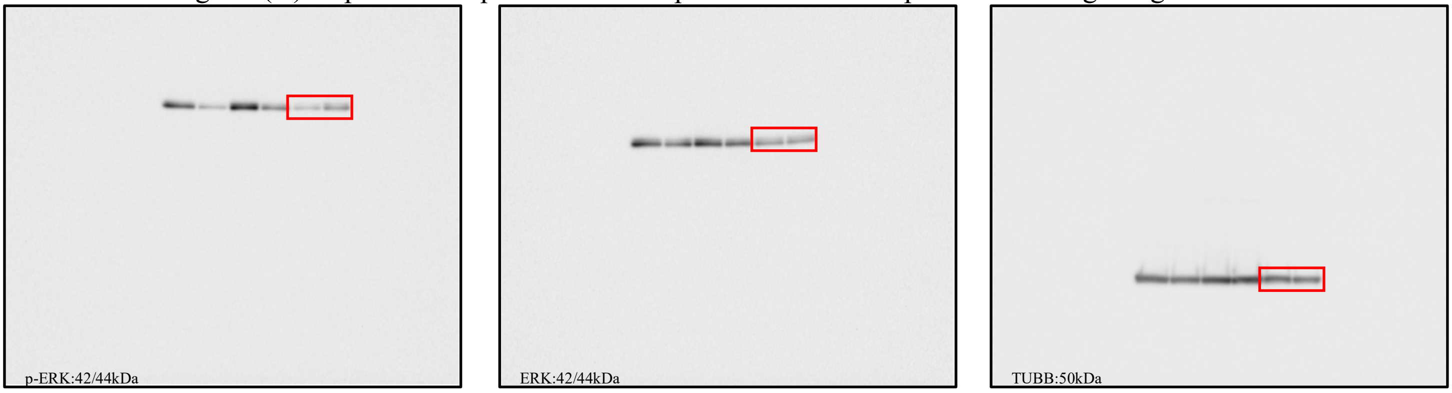

Figure2(A) Hep3B and LR7.5-3B Semi-quantification of p-ERK/ACTB was performed using Image J

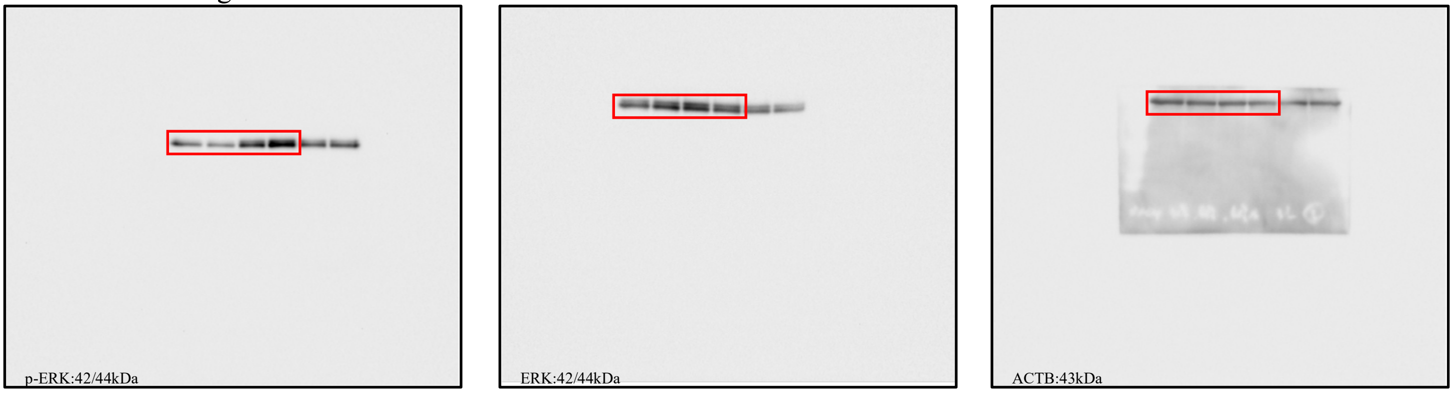

Figure2(B) JHH-4(7.5) Semi-quantification of p-ERK/TUBB was performed using Image J

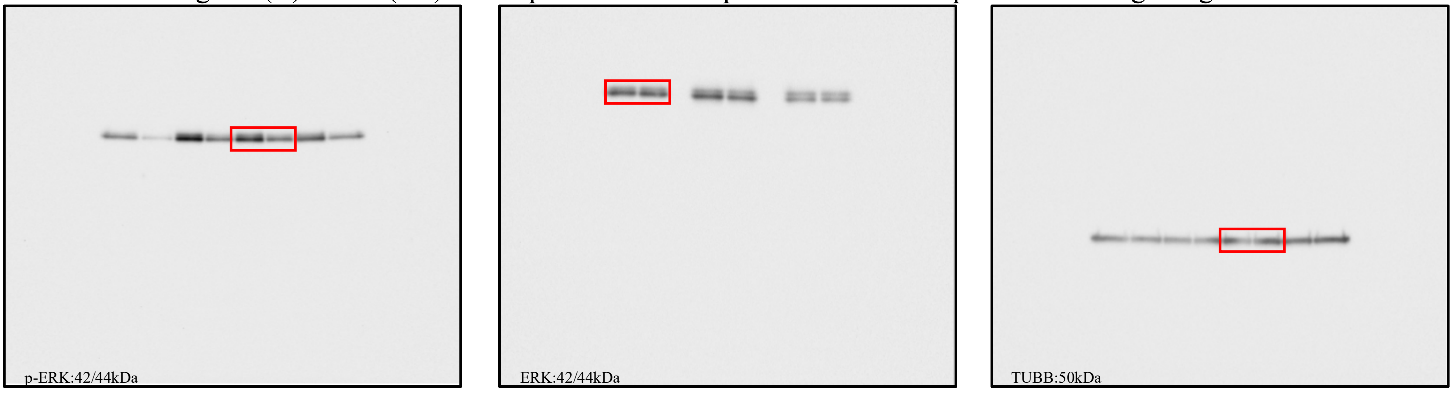

Figure2(B) Huh6(1.875) Semi-quantification of p-ERK/TUBB was performed using Image J

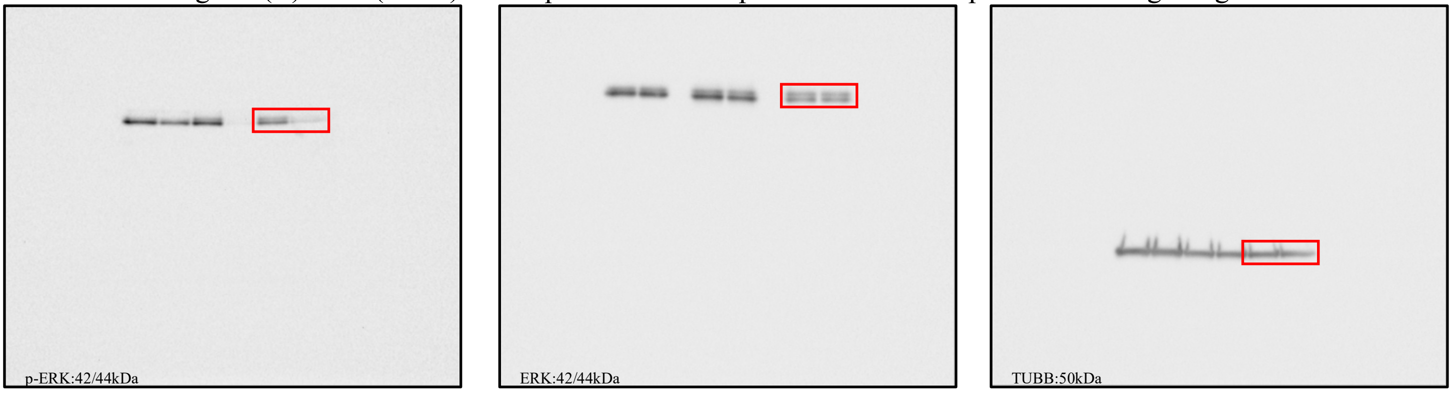

Figure S3

Figure3(E) 3B and LR Semi-quantification of p-EGFR/ACTB was performed using Image J

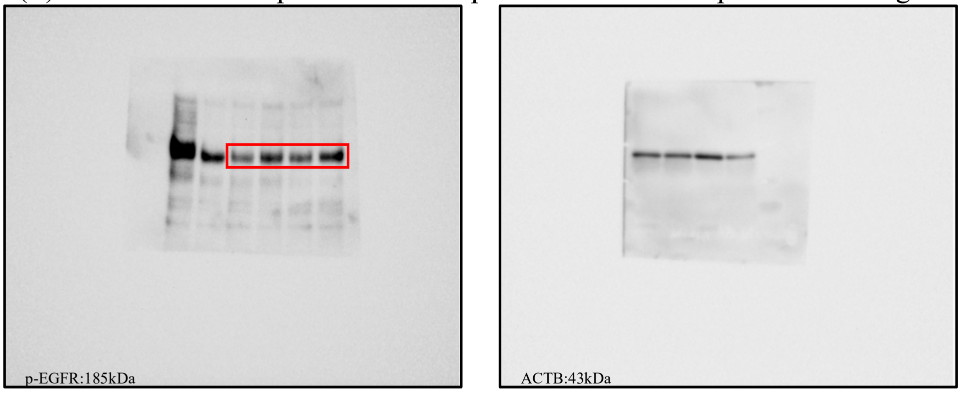

Figure3(F) 3B and LR Semi-quantification of p-INSR/ACTB was performed using Image J

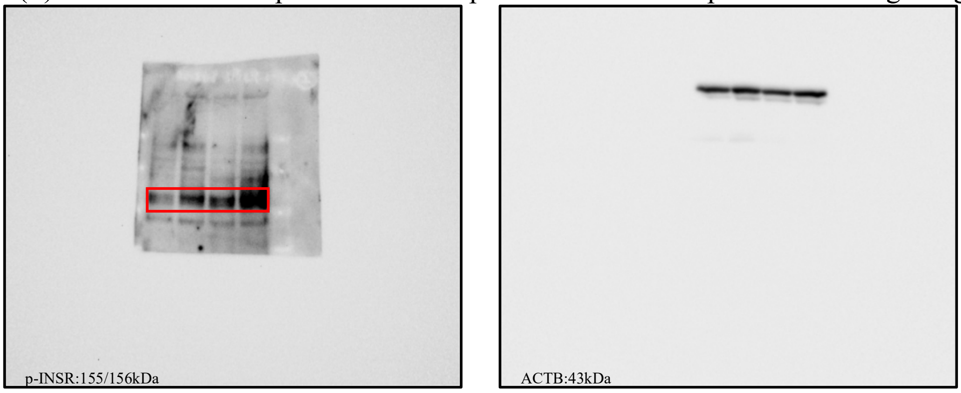

Figure4(A) Hep3B and LR7.5-3B Semi-quantification of p-ERK/ACTB was performed using Image J

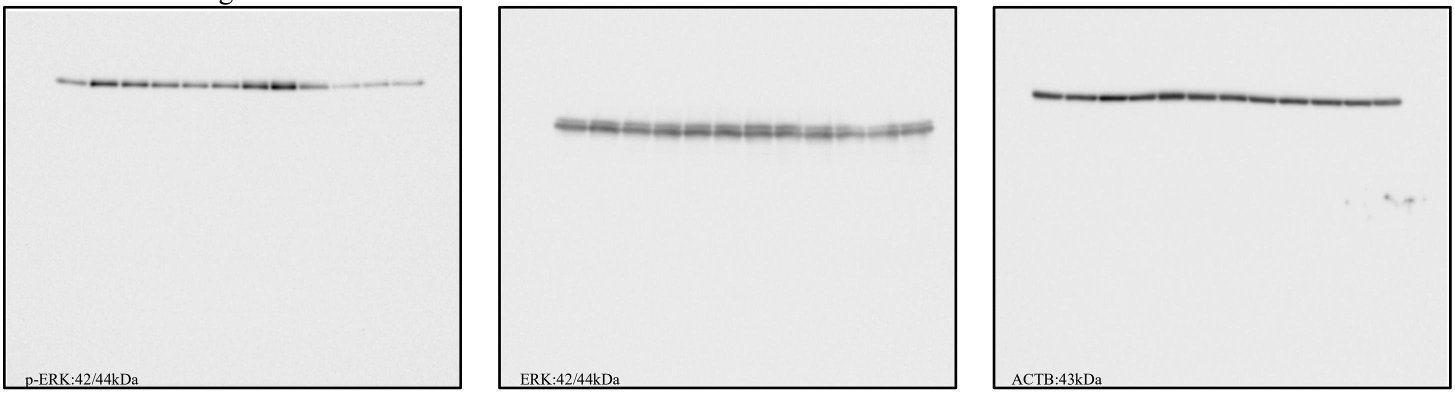

Figure4(D) Hep3B and LR7.5-3B Semi-quantification of p-ERK/ACTB was performed using Image J

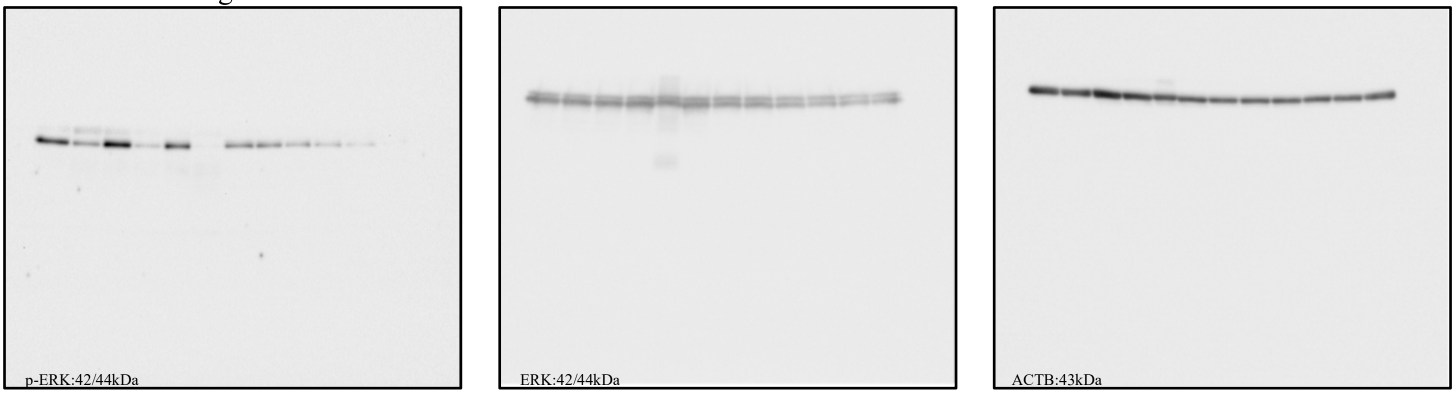

Figure S4

Figure5(A) Hep3B and LR7.5-3B Semi-quantification of p-ERK/ACTB was performed using Image J

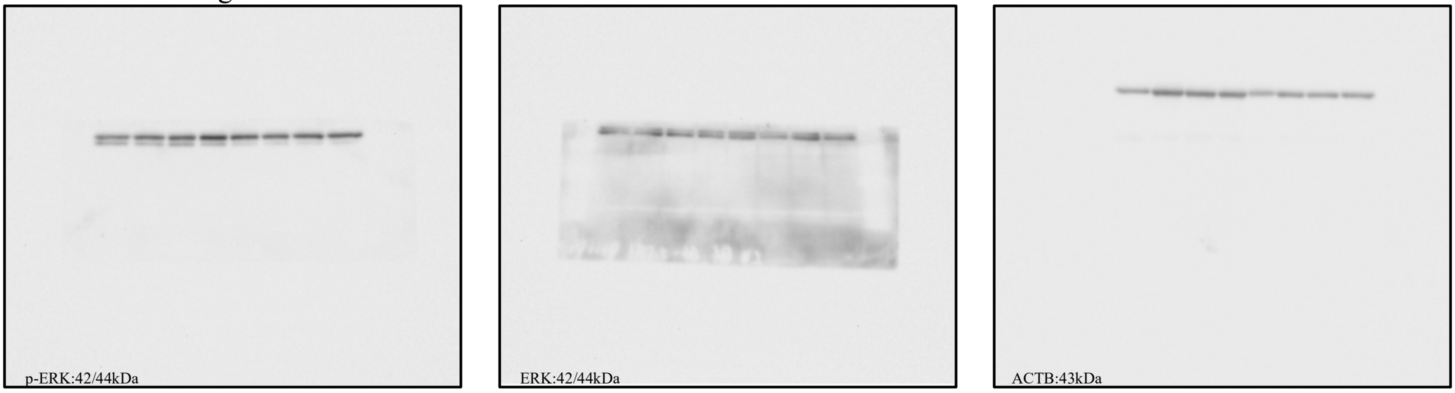

Figure5(D) Hep3B and LR7.5-3B Semi-quantification of p-ERK/ACTB was performed using Image J

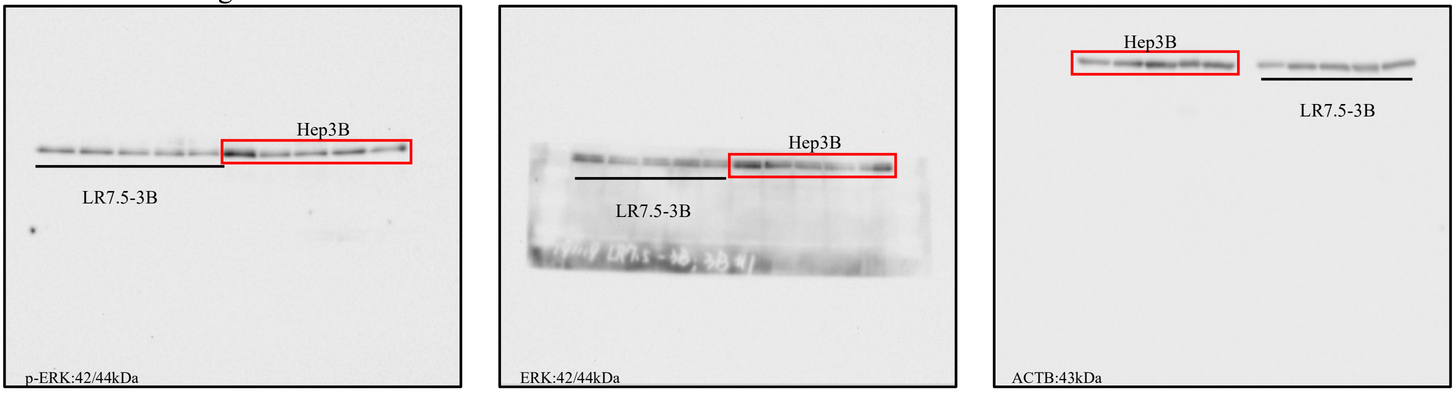

Figur6(C) Hep3B and LR7.5-3B Semi-quantification of p-ERK/ACTB and p-EGFR/ACTB was performed using Image J

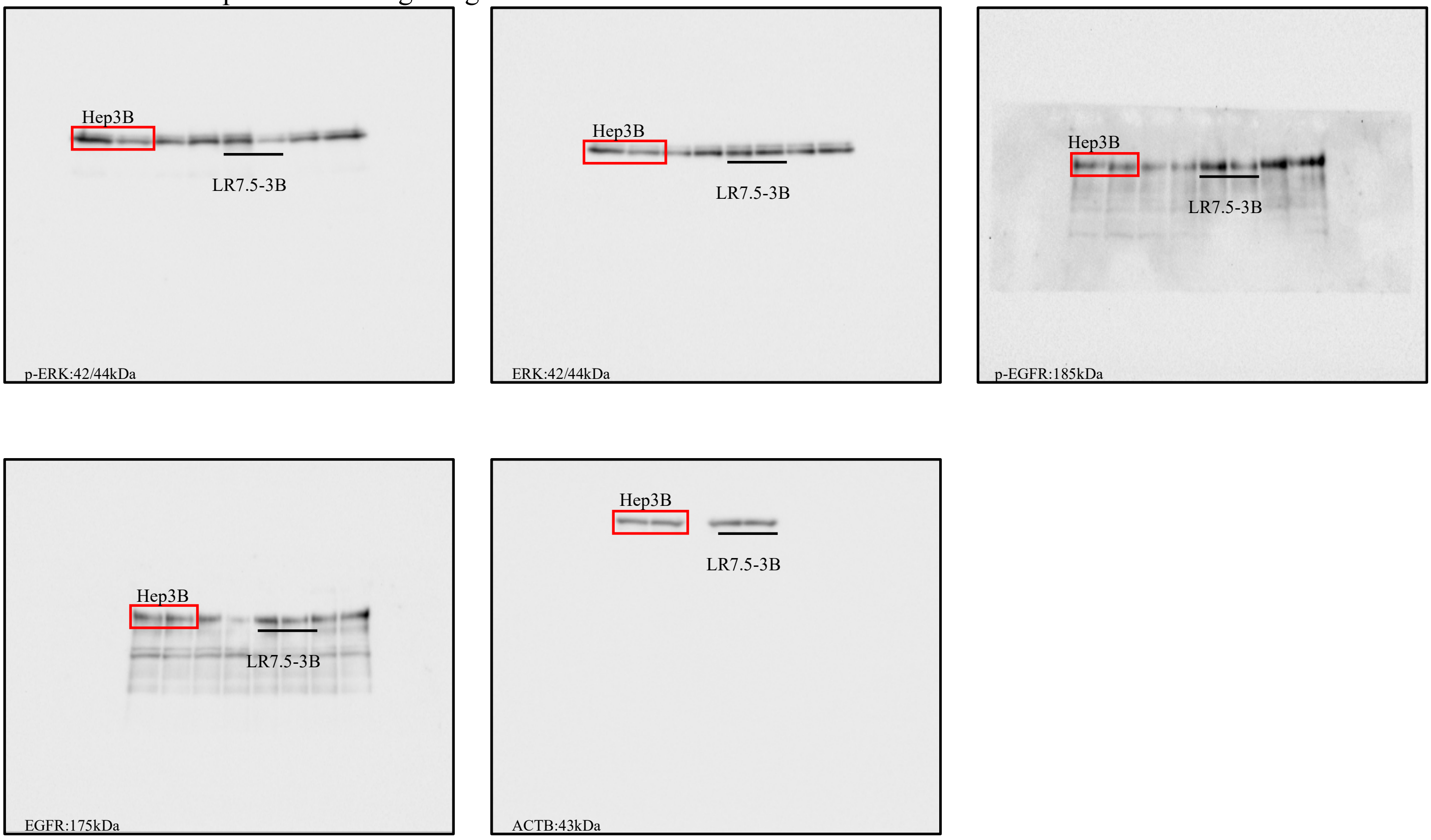

Figure S5

Figure S6. *The inhibition of EGFR or IGF1R by erlotinib or linstinib treatment,*

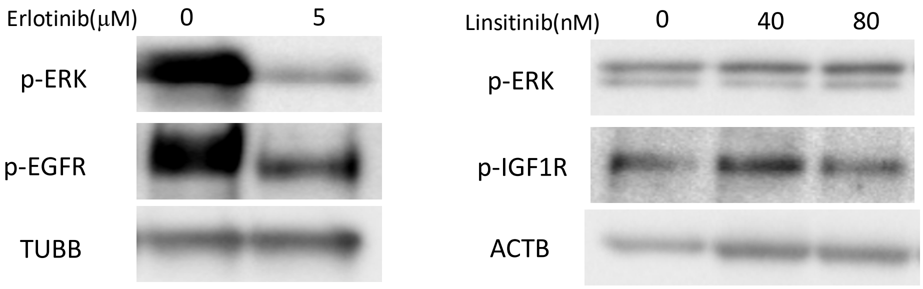

Figure S6

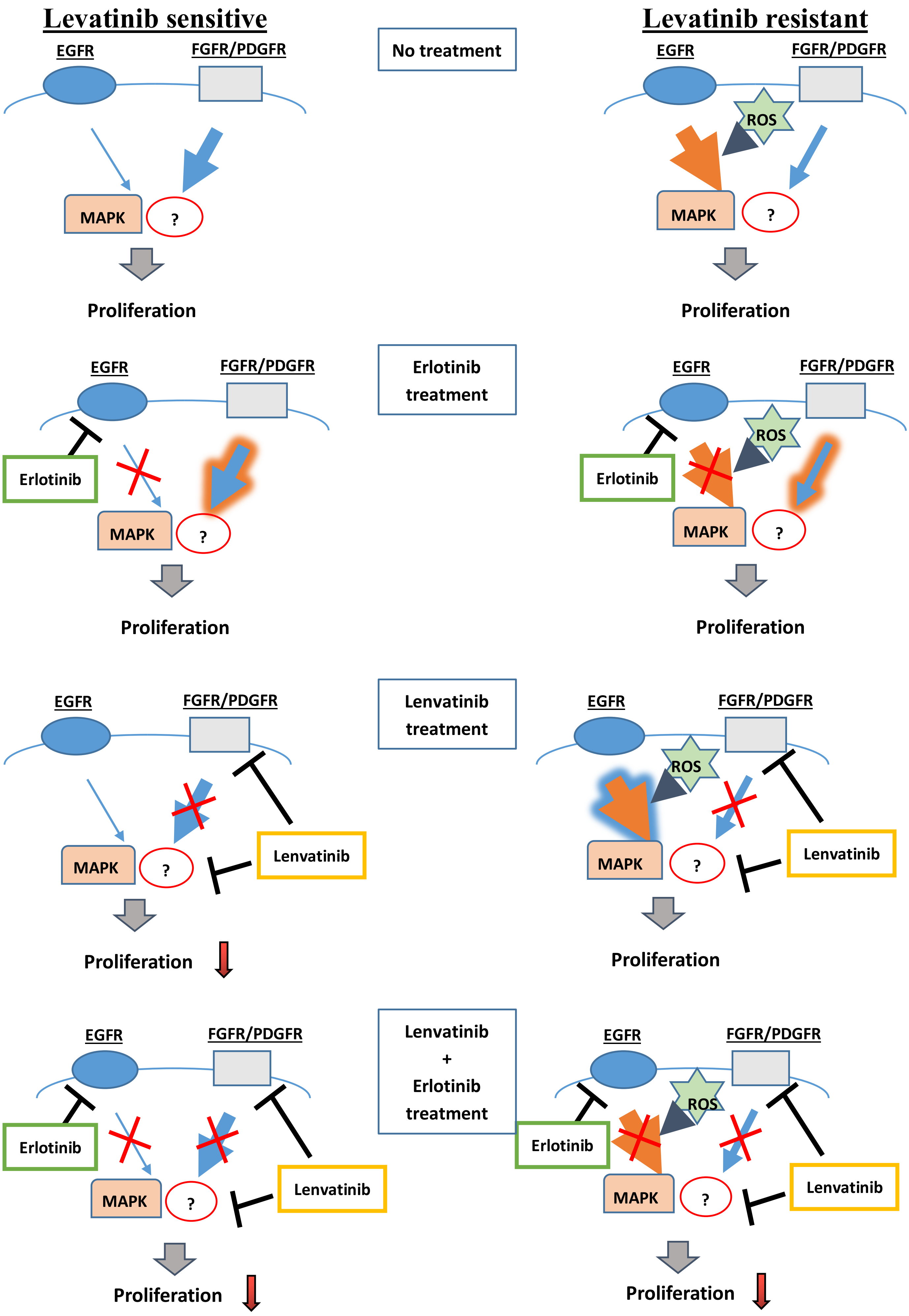

Levatinib sensitive
No treatment
Levatinib resistant
EGFR
FGFR/PDGFR
ROS
MAPK
?
Proliferation
Erlotinib
treatment
Erlotinib
Lenvatinib
treatment
Lenvatinib
Lenvatinib
+
Erlotinib
treatment

Figure S7
